# Variable activation in striatal subregions across components of a social influence task in young adult cannabis users

**DOI:** 10.1002/brb3.459

**Published:** 2016-04-22

**Authors:** Jodi M. Gilman, Sang Lee, John K. Kuster, Myung Joo Lee, Byoung Woo Kim, Andre van der Kouwe, Anne J. Blood, Hans C. Breiter

**Affiliations:** ^1^ Laboratory of Neuroimaging and GeneticsDepartment of PsychiatryMassachusetts General Hospital (MGH)CharlestownMassachusetts02129; ^2^ Athinoula A. Martinos Center in Biomedical ImagingDepartment of RadiologyMassachusetts General HospitalCharlestownMassachusetts02129; ^3^ Harvard Medical SchoolBostonMassachusetts02115; ^4^ Warren Wright Adolescent CenterDepartment of Psychiatry and Behavioral SciencesNorthwestern University Feinberg School of MedicineChicagoIllinois06011; ^5^ Mood and Motor Control LaboratoryMassachusetts General HospitalCharlestownMassachusetts02129; ^6^ Laboratory for Computational NeuroimagingDepartment of RadiologyMassachusetts General HospitalCharlestownMassachusetts02129

**Keywords:** Cannabis, marijuana, nucleus accumbens, peer groups, reward, social influence

## Abstract

**Introduction:**

Decades of research have demonstrated the importance of social influence in initiation and maintenance of drug use, but little is known about neural mechanisms underlying social influence in young adults who use recreational drugs.

**Methods:**

To better understand whether the neural and/or behavioral response to social influence differs in young adults using illicit drugs, 20 marijuana‐using young adults (MJ) aged 18–25, and 20 controls (CON) performed a decision‐making task in the context of social influence, while they underwent functional magnetic resonance imaging scans. A priori analyses focused on the nucleus accumbens (NAc), with post hoc analyses in the rest of the striatum. In this task, participants could choose to either follow or go against group influence.

**Results:**

When subjects applied social information to response choice selection (independent of following or going against group influence), we observed activation in the middle striatum (caudate), in the MJ group only, that extended ventrally into the NAc. MJ users but not CON showed greater activation in the NAc but not the caudate while making choices congruent with group influence as opposed to choices going against group influence. Activation in the NAc when following social influence was associated with amount of drug use reported. In contrast, during the feedback phase of the task we observed significant NAc activation in both MJ and CON, along with dorsal caudate activation only in MJ participants. This NAc activation did not correlate with drug use.

**Conclusions:**

This study shows that MJ users, but not CON, show differential brain activation across striatal subregions when applying social information to make a decision, following versus going against a group of peers, or receiving positive feedback. The current work suggests that differential neural sensitivity to social influence in regions such as the striatum may contribute to the development and/or maintenance of marijuana use.

## Introduction

Marijuana use is steadily increasing among young adults (Henry et al. 2003). Young adults may both initiate and continue to use marijuana in part due to social factors; simply, they may feel pressure to use because their peers are using. In a survey of 106 marijuana users (average age = 25.4), almost half of participants cited social pressure as a motive for using (Hartwell et al. 2012). This effect may be even more profound in younger adults, as young adults have been shown to be more vulnerable to peer influence than older adults (Gardner and Steinberg 2005). Peers play a pivotal role in introducing an individual to a drug (Clayton and Lacy 1982; Khavari 1993), and most drug use occurs in social and recreational settings (Terry‐McElrath et al. 2009). Young adults using marijuana may be more susceptible to peer influence than nonusing controls, and this vulnerability may be reflected in patterns of brain activity.

Little is known about neural mechanisms underlying social influence in the context of drug use. fMRI (Functional magnetic resonance imaging) studies have shown that structures such as the NAc (nucleus accumbens), amygdala, striatum, cingulate, anterior insula, and prefrontal cortex are activated when individuals follow peer influence, make a decision going against a peer group (Berns et al. 2005, 2010; Klucharev et al. 2009) or cooperate with others (King‐Casas et al. 2005). The NAc has emerged as an important region in social reward (Aharon et al. 2001). Research has shown that agreeing with peers increases NAc activity, whereas disagreeing decreases NAc activity (Zaki et al. 2011). These studies suggest that individuals process both the rewarding aspect of social stimuli and the congruence of social consensus with other people using traditional reward circuitry such as the NAc (Becerra et al. 2001; Breiter et al. 2001; Tom et al. 2007; Zaki et al. 2011). The increases and decreases in NAc activity observed in social influence studies are consistent with other work showing the NAc is one of the few brain regions showing positive and negative signal changes (Becerra et al. 2001; Breiter et al. 2001; Tom et al. 2007). In parallel to the social influence literature, altered NAc structure, function and neurotransmitter function has also been shown to be associated with cannabis use itself in humans (Filbey et al. 2009; DiNieri et al. 2011; Gilman et al. 2014; Smith et al. 2014; Yip et al. 2014; Ceccarini et al. 2015), paralleling animal literature showing a subset of brain regions implicated in social influence, such as the NAc, are structurally and functionally altered by THC exposure (e.g., Kolb et al. 2006; DiNieri et al. 2011). Therefore, the NAc has emerged as a region of interest at the intersection of cannabis use and social influence. Neuroimaging studies of social influence have not yet been conducted in populations using illicit drugs, nor have they been compared to reward‐related processing in populations using illicit drugs.

In this study, we investigated the neural correlates of social influence in young adults (age 18–25) using marijuana, and healthy nonusing controls. We evaluated activation to social influence in the NAc when subjects followed versus went against group influence, as well as control analyses regarding responding to influence in general and responding to reward‐related activity in the same region during feedback about task performance. We further evaluated if congruent/incongruent choice was associated with marijuana use measures. Young adults were the focus of this study because this age is a critical developmental stage in the initiation of drug use; mean age of onset of cannabis use is 17.7 years (Wittchen et al. 2008). This is an important group to study in order to understand the transition from recreational use to problem use, as younger initiation of drug use is a risk factor in itself for addiction (National Institute on Drug Abuse 2010).

One of the obstacles to studying the neuroscience of social influence is the absence of a well‐validated task. In a series of classical social psychology experiments in the 1950s (Asch 1951, 1952, 1956), Solomon Asch used a simple paper‐ and pencil line‐judgment task to demonstrate that individuals were likely to agree with peers even at the expense of accuracy. In this study, we developed a computerized version of this task that segregated stages of the decision‐making process and could be performed during fMRI scanning (Gilman et al. 2012). Our adaptation of the Asch experiment included an evaluation and action selection (i.e., “choice”) phase, a separate assessment of certainty about the decision selection, and a feedback phase. Given reports regarding NAc activation during social influence (Berns et al. 2005, 2010; Klucharev et al. 2009; Zaki et al. 2011), we hypothesized that social influence would be most salient during the “choice” phase, which in the current experiment, consisted of a participant either following or going against a group response. We expected, in particular, to observe NAc activation during the phase in which participants made choices that were consistent with group influence. We further hypothesized, given the peer pressure literature in addiction (Clayton and Lacy 1982; Khavari 1993; Hartwell et al. 2012), that during the choice phase, young adults using marijuana would (1) be more likely than nonusing controls to follow group information, (2) show hyperactivation of brain regions underlying social reward such as the NAc, and (3) demonstrate a relationship between NAc activation during choice and marijuana consumption. In order to evaluate whether potential differences were specific to the choice phase, we also investigated whether any differences observed during the feedback phase of outcome evaluation (e.g., monetary reward) were related to marijuana use. In addition to a region‐of‐interest analysis of the NAc, we also performed a whole‐brain analysis in order to investigate whether other brain regions differed between marijuana users and nonusing controls during response choices made after social influence in general (e.g., combining following and going against group influence). These other brain regions included regions of the caudate, along with insula, cingulate, and prefrontal cortex as reported by others for social influence (Berns et al. 2005, 2010; Klucharev et al. 2009).

To further evaluate whether drug exposure affected our findings, we performed a *post hoc* analysis relating functional imaging findings to structural differences associated with marijuana exposure. Specifically, we investigated whether GMd (gray matter density) in the NAc, measured with voxel‐based morphometry, was associated with activation to social influence. On the basis of animal studies showing morphometric abnormalities in the NAc but not the striatum (Kolb et al. 2006), and human studies showing similar effects in the NAc (Gilman et al. 2014), we hypothesized that GMd in this region would possibly be associated with (1) magnitude of fMRI activation in response to social influence (i.e., following vs. going against group influence), and (2) indices of MJ use.

## Method

### Participants

Participants in this study were 40 young adults, age 18–25; 20 (10 women) who regularly used marijuana (MJ), and 20 (10 women) controls (CON). MJ and CON were matched on age, gender, handedness, race, and years of education (Table 1). MJ used marijuana at least once a week, but were not dependent, according to a Structured Clinical Interview for the DSM‐IV (SCID) (First et al. 2002); marijuana dependence was exclusionary because evidence suggests that peer influence may have more of an impact in recreational users than it would in substance‐dependent patients, where drug‐taking may become less social and more habitual (Wise 1996). MJ were not excluded if they had used other illegal drugs in the past; however, they were excluded if they ever met abuse criteria for any drug other than marijuana. CON participants had not used marijuana in the past year, and had used marijuana on less than five occasions in their lifetime. Time‐line follow‐back methods were used to quantify marijuana and alcohol exposure as described in the Procedure section.

**Table 1:** Participant demographics

		CON (*n* = 20)	MJ (*n* = 20)	*P* value
Gender		10 M/10 F	10 M/10 F	N/A
Age		20.4 (1.7)	21.4 (2.0)	0.11
Years of Education		14.2 (3.3)	12.7 (4.8)	0.25
STAI^a^	State	28.9 (7.94)	27.7 (7.38)	0.65
	Trait	29.8 (7.32)	29.5 (5.56)	0.89
HAM‐D^b^		0.65 (1.31)	1.15 (1.39)	0.25
		Range [0–3]	Range [0–3]	
TIPI^c^	Extroversion	10.15 (2.91)	10.65 (2.41)	0.56
	Agreeableness	10.35 (2.48)	10.20 (2.21)	0.84
	Conscientiousness	12.05 (2.01)	11.15 (2.54)	0.22
	Emotional Stability	10.95 (2.70)	10.50 (2.86)	0.61
	Openness	11.70 (1.92)	12.37 (1.57)	0.24
*Substance use*				
Alcohol	# Alcoholic Drinks/Week	2.84 (2.57)	5.13 (4.73)	0.08
Cigarettes	# Occasional cigarette smokers	0	8	N/A
	# Daily cigarette smokers	0	1	N/A
Marijuana	# MJ Use Days/week	N/A	3.96 (2.25)	N/A
	# MJ Joints per week	N/A	11.62 (9.76)	N/A
	Median # MJ Joints per week	N/A	9.62	N/A
	Age of Onset (years)	N/A	16.35 (2.24)	N/A
	Duration of Use (years)	N/A	6.34 (3.53)	N/A
	Length of Abstinence before scan (days)	N/A	2.26 (2.08) Range [1–9 days]	N/A

CON, controls; MJ, marijuana users.

All values are expressed in means and standard deviations.

a State Trait Anxiety Inventory Form (Spielberger 2010);

b Hamilton Depression Rating Scale (Hamilton 1960);

c Ten‐Item Personality Inventory (Gosling et al. 2003).

Both groups were medically healthy with self‐reported normal or corrected‐to‐normal vision, and did not meet DSM‐IV criteria for any current or lifetime Axis I disorders. All participants completed the AUDIT (Alcohol Use Disorder Identification Test) (Saunders et al. 1993) to check for problem drinking; any participant scoring above an 8, indicative of hazardous drinking, was excluded. All participants gave written informed consent to a protocol approved by the Partners Human Research Committee Institutional Review Board.

### Procedure

Participants completed screening and testing during one study visit. All MJ were asked to refrain from using substances on the day of the study. We performed a urine drug screen that tested for cannabis, amphetamines, cocaine, barbiturates, methamphetamines, benzodiazepines, codeine, morphine, and ethanol. Since THCCOOH, the main secondary metabolite of tetrahydrocannabinol (THC), can be detected in urine several weeks after last use (Fraser et al. 2002), we ensured that no participant exhibited overt signs of intoxication, based on a four‐item marijuana intoxication scale developed in our laboratory that was designed to assess four signs of acute intoxication (Karschner et al. 2011): increased resting heart rate (>100 beats per minute), congestion of the conjunctival blood vessels (red eyes), slowed speech response, and giddiness. No MJ participants were excluded based on these criteria.

MJ participants completed a time‐line follow‐back (Sobell et al. 1986) asking them to indicate, for the past 90 days, the days that they smoked marijuana. They were given a calendar, and asked to check off the days on which they used marijuana, to the best of their ability, focusing on patterns of use. They were also asked how many separate times in a day they used, and how many joints (or joint equivalents) they consumed per smoking occasion. All participants (MJ and CON) also completed a time‐line follow‐back for alcohol use (Sobell et al. 1986), asking them to detail their drinking behavior in the past 90 days. All participants completed the State Trait Anxiety Inventory Form (STAI) (Spielberger 2010) to assess anxiety symptoms, the Hamilton Depression Rating Scale (HAM‐D) (Hamilton 1960) to assess mood issues, and the Ten‐Item Personality Inventory (Gosling et al. 2003) to assess personality characteristics. If group differences on any alcohol or clinical/personality measures showed a trend or significant effect, we used this measure in separate regression analyses against the imaging data.

### Social influence task design

**Figure 1:**
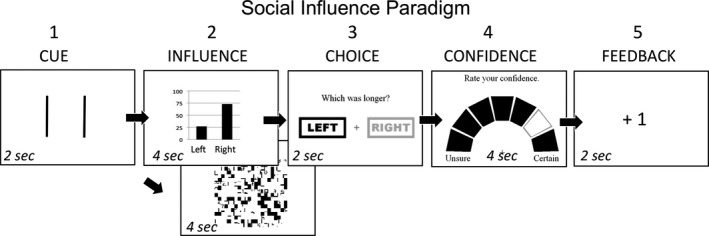
Schematic of social influence task. This task consisted of five events, described in “Methods,” which represented discrete phases of decision making. Event 1 (Cue) and Event 2 (Influence) represented evaluation; Event 3 (Choice) represented action selection; Event 4 (Confidence) represented a separate assessment of certainty about the decision selection; Event 5 (Feedback) represented evaluation of outcomes. In Event 2 (Influence), the participant could either be presented with a graph of “peer” responses (top), or a scrambled graph without peer information (bottom).

The social influence task was designed to measure an individual's likelihood of following group decisions or making independent choices in a visual discrimination task. Each trial consisted of five events, shown in Figure 1. In **Event 1 (Cue)**, the participant saw a cue, consisting of two lines, and was asked to judge which line – the left or the right – was longer. In half of the trials, the task was “easy” (i.e., participants can easily tell which was longer, yielding <5% error rate); and in the other half, the task was “hard” (yielding ≈ 50% error rate). We included easy and hard trials in order to determine whether participants would follow the group on easy trials even when they were sure of the correct answer; this was an important component of the initial Asch experiment (Asch 1951, 1952, 1956). In **Event 2 (Influence)**, responses of a fictitious “group” were revealed to the participant in the form of a bar graph indicating the percentage of the group that chose “left” or “right” as the longer line. Participants were told that the graph represented the responses of others who had done the experiment. (Studies have shown that peer influence can have an effect even when peers are not physically present (Berns et al. 2010)). In 50% of trials, the group responses were correct (i.e., the line on the left was longer, and the “group” recommended “left”), and in 50%, the “group” was incorrect (i.e., the line on the left was longer but that “group” recommended “right”). As a control condition, in 50% of the trials, participants saw a noise image, based on scrambling of all graphs used in Event 2 of the task, which did not provide any group information (Fig. 1, lower image). Trial types were presented in a random order. In **Event 3 (Choice)**, the participant completed their judgment of which line segment was longer (“Left” or “Right”) and selected their choice based on this judgment using a button box. In **Event 4 (Confidence)**, the participant was asked to rate his/her confidence in that judgment on a Likert‐like scale. Next, in **Event 5 (Feedback)**, the participant was told if he/she was correct on that trial. If the participant made the correct judgment, he/she received a point. Next, there was an intertrial interval consisting of a fixation point, which was presented for 1–5 sec. An interevent jitter (nonintegers between 1 and 3 sec) was introduced which allowed us to separate each event of a trial for analysis. The five events in each trial corresponded to current schemas in judgment and decision making as follows: **Events 1** and **2** related to option identification and valuation phases of decision making, **Event 3** related to the action selection phase (i.e., “choice”), **Event 4** related to a separate judgment (about certainty) and potentially included anticipation of an outcome from a choice, and **Event 5** related to the phase of decision making in which an outcome was evaluated with feedback. There were 96 trials in total, split into three runs of 32 trials each. Each trial took 16 sec. Breaks were built in after each run to prevent fatigue.

The task took approximately 30 min to complete. A monetary reward was given at the end of the experiment based on the number of points received. Specifically, points received were translated into money as follows: over 80 points = $20, 70–79 points = $15, 60–69 points = $10, and 20–59 points = $5. After the experimental session was completed, all subjects went through a complete debriefing in which the study staff fully explained the purpose of the study, and explained the need for deception.

### Behavioral analysis

We conducted a two‐way ANOVA (SPSS, Version 19, IBM Corp., Armonk, NY) to assess differences in behavior between groups. The independent variable was group (CON, MJ), and the dependent variable was choice (congruent, incongruent) during Event 3, which was the main focus of this study; a congruent choice was one in which the participant's choice in Event 3 (Choice) matched the group recommendation in Event 2 (Influence), and an incongruent trial was one in which the group recommended one option in Event 2, and the participant chose the opposite during Event 3. Because some participants missed some responses (i.e., they did not respond in the allotted time), the number of choices was divided by the total number of responses for each participant, in order to normalize their score. We also conducted ancillary ANOVAs to investigate whether the confidence ratings (i.e., **Event 4**) during each trial type differed between groups, and whether or not points and money received for **Event 5** differed between groups. Where we found a significant effect of either independent variable, or a significant interaction, we then followed the ANOVA with post hoc tests (Tukey) to assess differences among task conditions.

We also conducted two‐way ANOVAs to assess differences between groups in reaction time to the choice event (independent variable was group (CON, MJ), dependent variables were difficulty (easy, hard trials), and choice type (congruent, incongruent choices). We also conducted within‐group one‐way ANOVAs to examine potential differences in reaction time across four possible conditions (e.g., congruent easy, congruent hard, incongruent easy, and incongruent hard trials). Where we found a significant *F* value (*P* < 0.05), we then followed the ANOVA with post hoc tests (Tukey) to assess differences among task conditions.

Correction for the number of ANOVAs run was the Bonferroni correction *P* < 0.05/5 = 0.01), with the exception of the congruent/incongruent choice comparisons (*P* < 0.05), which was the a priori focus of this study.

### Acquisition and analysis of neuroimaging data

Participants were scanned using a 3 T Siemens (Erlangen, Germany) Trio scanner with a 32 channel head coil at the Martinos Center for Biomedical Imaging. Whole‐brain T1‐weighted 1 mm isotropic structural scans were collected using a 3D multiecho MPRAGE sequence (176 sagittal slices, 256 mm FoV, TR 2530 msec, TI 1200 msec, 2x GRAPPA acceleration, TE 1.64/3.5/5.36/7.22 msec, BW 651 Hz/px, T_acq_ 6:03 min) (van der Kouwe et al. 2008). Functional scans were collected using a 2D gradient echo EPI sequence (31 slices, 3 mm thick, 0.6 mm gap, 216 mm FoV, 3 mm^2^ in‐plane resolution, TR 2 sec, TE 30 msec, BW 2240 Hz/px). All acquisitions were automatically positioned using AutoAlign (van der Kouwe et al. 2005).

Functional magnetic resonance imaging data processing was carried out using FEAT 1 (FMRI Expert Analysis Tool) Version 5.98, part of the FSL fMRI processing stream (FSL's Software Library, www.fmrib.ox.ac.uk/fsl). Registration was done in two steps; first, each participant's functional and structural scans were registered using FLIRT (FSL's linear registration tool), and then these scans were registered to high‐resolution structural and standard space images using both FLIRT and FNIRT (FSL's nonlinear registration tool), (Jenkinson and Smith 2001; Jenkinson et al. 2002) so that each participant's brain was registered to the ICBM152 T1 template (Chau and McIntosh 2005). Motion outliers were removed using the FSL tool “fsl_motion_ouliers” (http://fsl.fmrib.ox.ac.uk/fsl/fslwiki/FSLMotionOutliers). In addition, the following preprocessing was applied; nonbrain removal using BET (FSL's Brain Extraction Tool) (Smith 2002); spatial smoothing using a Gaussian kernel of FWHM 5 mm; grand‐mean intensity normalization of the entire 4D dataset by a single multiplicative factor; high pass temporal filtering (Gaussian‐weighted least‐squares straight line fitting, with sigma = 50 sec).

Analysis involved an ROI (region‐of‐interest) analysis for our a priori region, the NAc, and whole‐brain voxel‐by‐voxel analysis for all other regions. For the ROI‐based analysis of fMRI data, individual parameter estimates for the NAc were extracted using the FSL program featquery (http://fsl.fmrib.ox.ac.uk/fsl/fsl4.0/feat5/featquery.html). Activation signal was extracted from each participant using the following steps: (1) the signal at each voxel was converted to a (percentage) deviation from the mean for that voxel across the entire time series, (2) the signal was averaged by stimulus type and spatially translated into MNI space, and (3) anatomical masks were designated consisting of the volume of interest through which each individual participant's data were extracted. All masks were parcellated from the ICBM152 T1 brain at the MGH CMA (Center for Morphometric Analysis), using validated landmarks for the NAc (Breiter et al. 1997; Gasic et al. 2009; Perlis et al. 2008). ROIs of the left and right NAc were chosen a priori based on regions previously implicated in social decision making (Aharon et al. 2001; Klucharev et al. 2009; Zaki et al. 2011). After extracting data from these ROIs from each individual, values were entered into two‐way ANOVAs to investigate whether there were significant main effects or interactions for the following conditions. (A) One ANOVA evaluated interactions between group (MJ, CON), and influence in general (congruent and incongruent choice together, noise images) for **Event 3.** (B) Another ANOVA evaluated interactions between group (MJ, CON), and choice (congruent, incongruent) for **Event 3**. (C) A third ANOVA evaluated interactions between group (MJ, CON), and feedback (gain, no‐gain) for **Event 5**. Given the ANOVA for (B) reflected the primary focus for this experiment and our a priori hypotheses, this result was corrected for evaluation of the left and right NAc separately, or *P* < 0.05/2 = 0.025. The other two control analyses were corrected for the number of ANOVAs run (two conditions, left and right NAc each condition, or *P* < 0.05/4 = 0.0125). Values were also regressed against drug use measures and any other alcohol or clinical/personality measures showing trend effects, in order to investigate whether activation was associated with amount/frequency of marijuana use or these other measures. These analyses, like the control analyses, were subject to Bonferroni corrections for the number of analyses run; given many of the MJ and alcohol use measures correlated with each other and were thus not independent measures, all results are listed which met the correction for uncorrelated measures (*P* < 0.05/4 = 0.0125 for left and right NAc, MJ and alcohol).

For the whole‐brain, voxel‐based analyses looking at brain regions outside the NAc, including regions of the striatum contiguous with the NAc, we performed two steps. First, each participant's time series data were fit using a linear signal model with nine regressors of interest, and six movement regressors of no interest. Regressors were as follows: (1) response to cue, (2) response to scrambled graph, (3) response to influence, (4) response to choice, when participant agreed with group influence (“Congruent”), (5) response to choice, when participant went against with group influence (“Incongruent”), (6) response to choice, after participant viewed a scrambled group (“No Influence”), (7) response to rating confidence, (8) response to positive feedback (gaining a point), and (9) response to negative feedback (not gaining a point). Time series statistical analysis was carried out using FILM with local autocorrelation correction (Woolrich et al. 2001). *Z* (Gaussianized T/F) statistic images were thresholded using clusters determined by *Z* > 2.6 and a (corrected) cluster significance threshold of *P* = 0.05 (Worsley 2001) in the first level (individual subject) analysis. Next, higher level group analysis was carried out using FLAME (FMRIB's Local Analysis of Mixed Effects) stage 1 and stage 2 (Beckmann et al. 2003; Woolrich et al. 2004, 2009). Clusters larger than 20 voxels at an individual voxel threshold of *P* < 0.005 were considered significant (Lieberman and Cunningham 2009). This cluster size and threshold was chosen based on simulations demonstrating that combined intensity and cluster size thresholds such as *P* < 0.005 with a 20 voxel extent produce a desirable balance between Types I and II error rates for cognitive (e.g., nonmotor) studies (Lieberman and Cunningham 2009).

Our whole‐brain, voxel‐based analyses consisted of a random effects analysis of two primary contrasts of interest: (1) Choice after Influence versus Choice after No Influence (Scrambled), which isolated areas of the brain that responded to group information in general, and (2) Congruent versus Incongruent Choice, which isolated brain areas that were more responsive to following than dissenting from group information. Additionally, we examined (3) Positive Feedback (Gaining a point) versus Negative Feedback (Not gaining a point), in order to determine whether there were any observable differences during the feedback phase of decision making between groups. Other regressors were necessary for modeling the paradigm, but were not directly relevant to the hypotheses of the study.

### Voxel‐based morphometry analysis

Structural data were analyzed with the standard processing stream of FSL‐VBM (http://fsl.fmrib.ox.ac.uk/fsl/fslwiki/FSLVBM), an optimized VBM protocol carried out with FSL tools, as used in a previous publication, with a partially overlapping cohort (Gilman et al. 2014). First, structural images were brain‐extracted and gray matter‐segmented before being registered to the 2 mm MNI 152 standard space using nonlinear registration (Andersson et al. 2007) The resulting images were averaged and flipped along the *x*‐axis to create a left‐right symmetric, study‐specific gray matter template. Second, all native gray matter images were nonlinearly registered to this study‐specific template and “modulated” to correct for local expansion (or contraction) due to the nonlinear component of the spatial transformation. The modulated gray matter images were then smoothed with an isotropic Gaussian kernel with a sigma of 3 mm. Data were extracted from ROIs using validated landmarks and anatomical masks of the left and right NAc parcellated from the ICBM152 T1 brain at the MGH CMA (Breiter et al., 1997; Gasic et al. 2009; Perlis et al. 2008); these ROIs were identical to those in which fMRI data were extracted. The gray matter density (GMd) values thus extracted were then compared between groups and used in regression analyses against (1) fMRI signal from the same regions, (2) MJ use, and (3) any other clinical/personality measure showing at least trend effects between groups. The GMd values thus extracted were then compared between groups and used in regression analyses against (1) fMRI signal from the same regions, (2) MJ use, and (3) any other clinical/personality measure showing at least trend effects between groups.

## Results

### Participant characteristics

CON and MJ were not different in gender, age, years of education, handedness or race; they also did not differ on any of the behavioral questionnaires (STAI, HAM‐D, TIPI) (Table 1). MJ reported drinking a greater number of alcoholic drinks per week than CON, though the groups were not significantly different (*P* = 0.08).

### Behavioral results

CON and MJ were not significantly different in task performance. For easy trials, performance accuracy was 0.99 for CON (SD = 0.02) and 0.99 for MJ (SD = 0.024). For hard trials, accuracy was 0.56 for CON (SD = 0.1) and 0.51 for MJ (SD = 0.06). The amount of points earned and money received by CON and MJ was not significantly different (all *P* > 0.1).

**Figure 2:**
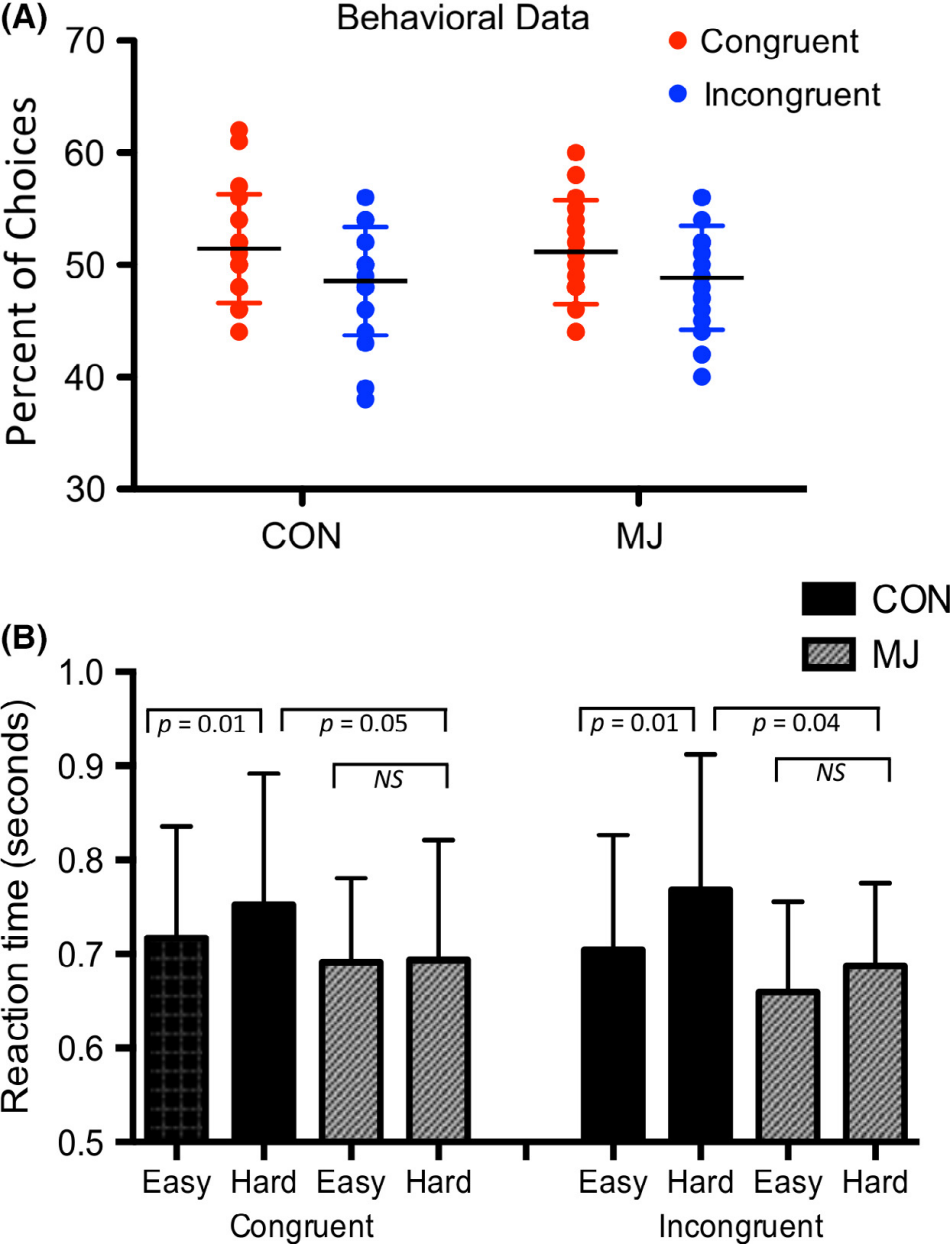
Behavior in social influence task. (A) A two‐way ANOVA of choice (congruent, incongruent), by group (CON, MJ) demonstrated a significant effect of choice. There was no group effect or interaction, demonstrating that both CON and MJ were more likely to make congruent than incongruent choices. Participants were more likely to make congruent than incongruent choices during hard, but not easy trials. (B) There were no group differences in reaction time overall (across all trial types). Within CON, there was a difference across the four trial types; CON had significantly longer reaction times during hard compared to easy trials for both congruent and incongruent choices. There were no differences within the MJ group across trial types. CON had greater reaction times than MJ during the hard trials, for both congruent and incongruent choices.

Both CON and MJ were more likely to make congruent than incongruent choices (*F* (1,38) = 6.01, *P* = 0.016), but there were no differences between groups (Fig. 2A). Participants were more likely to make congruent than incongruent choices during hard (*F* (1,38) = 4.84, *P* = 0.031), but not easy trials. Both groups rated higher confidence following easy than hard trials (*F* = 36.28, *P* < 0.001), but there were no group differences, and there were no differences in confidence ratings between congruent and incongruent choices (all *P* > 0.1). Neither CON nor MJ ever followed group influence on easy trials when the group was incorrect. The amount of points earned and money received by CON and MJ was not significantly different (all *P* > 0.01).

There were no group differences in reaction time overall (across all trial types). CON had significantly longer reaction times during hard compared to easy trials for both congruent and incongruent choices (*F* (3, 18) = 4.5, *P *=* *0.015; Fig. 2B). There were no differences within the MJ group across trial types. To facilitate future hypothesis generation, we evaluated post hoc tests and found there were no group differences in reaction time during easy trials, but during hard trials, CON had greater reaction times than MJ for both congruent (*P *=* *0.049) and incongruent (*P *=* *0.040) choices (Fig. 2B).

### Neuroimaging results

#### Activation to choice following social influence versus choice following NO influence (scrambled graphs)

**Figure 3:**
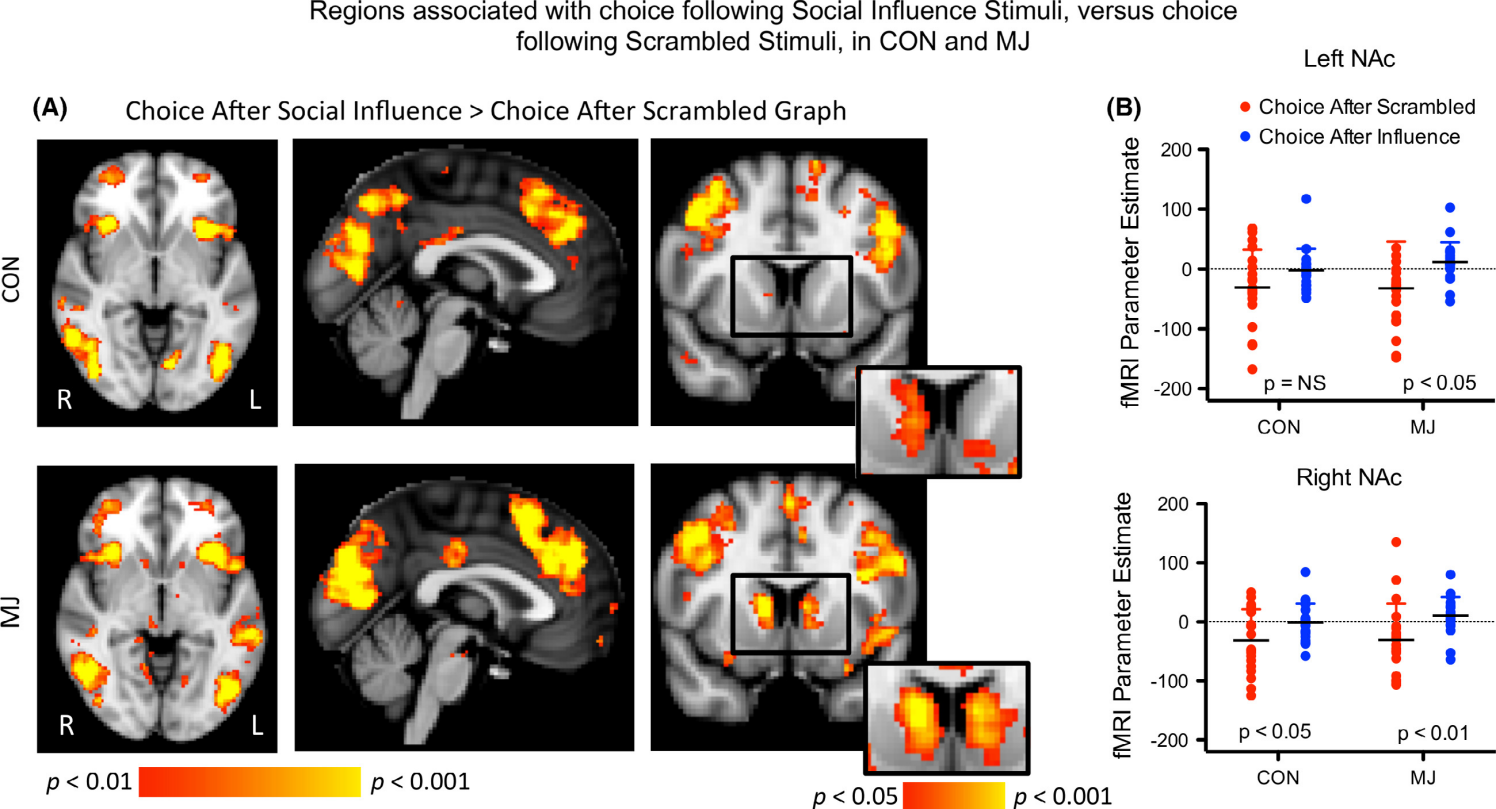
Activation during the choice phase following social influence versus noise stimuli. (A) The contrast of choice after social influence compared with choice after no influence activated a wide network of structures in both groups. Images are thresholded at *P* < 0.01, uncorrected. Notably, at this threshold, the caudate (designated by a square on the axial slices) only activated in the MJ group. When images were thresholded at *P* < 0.05 (illustrated at the bottom right of axial images), subthreshold signal change is observed in this region in CON, and the cluster extended into the NAc in both groups. (B) Scatterplot of NAc values based on ROI Analysis. In the left NAc (top), there was a significant effect of choice across both groups indicating greater activation during a choice following group influence than during a choice following a scrambled graph. Post hoc tests showed that there was significantly greater activation after influence than no influence in MJ (*P* < 0.05), but no difference in CON. In the right NAc, there was also a significant effect of choice across groups; Post hoc tests showed that there was significantly greater activation after influence than no influence in both MJ (*P* < 0.01) and CON (*P* < 0.05).

For this positive control analysis, the following brain imaging results refer to changes in activity during the “Choice” period, which was defined as the 2‐sec period during which the participant made a button press (i.e., the decision/response phase; Event 3 in Fig. 1) after seeing the peer influence. This contrast evaluated activity when participants made a choice (1) after receiving information from graphs showing peer responses, as compared to (2) after viewing scrambled graphs with no peer information (Fig. 3A and B). This analysis included congruent and incongruent trials together, and thus only determined if social information was being processed, and was not one of our a priori hypotheses relating to following versus going against group influence,

We evaluated effects in our a priori region, the NAc, by extracting averaged fMRI data (parameter estimates) from left and right NAc. In the left NAc (top), there was a significant effect of choice across both groups (*F* (1,38) = 9.59, *P* = 0.004), indicating a positive difference during a choice following group influence than during a choice following a scrambled graph (Fig. 3B). Post hoc tests showed that there was a significantly higher parameter estimate after influence than no influence in MJ (*t* = 1.7, *P* < 0.05), but no difference in CON. In the right NAc, there was also a significant effect of choice across groups (*F* (1,38) = 15.5, *P* < 0.001) (Fig. 3B). Post hoc tests showed that there was a significantly higher parameter estimate after influence than no influence in both MJ (*t* = 3.2, *P* < 0.01) and CON (*t* = 2.4, *P* < 0.05).

With the whole‐brain, voxel‐based analysis, both CON and MJ activated a broad network of brain regions associated with social cognition and decision making, including frontal structures such as the frontal pole, anterior cingulate, dorsolateral prefrontal cortex (DLPFC), middle and inferior frontal gyri (Table 2, Fig. 3A). Structures such as the anterior cingulate and insula were activated in both groups, though MJ participants showed larger activation volumes. Caudate activation was only observed in the MJ group. When images were thresholded at *P* < 0.05, subthreshold signal change was observed in the NAc in both groups (along with subthreshold signal change in CON), and signal change in the NAc was clearly contiguous with caudate activation, where the peak of the cluster was localized (Fig. 3A). In a direct comparison between groups, MJ showed significantly greater activation than CON in the left frontal pole, left superior frontal gyrus, and left superior parietal lobule.

**Table 2:** Activation to choice following social influence versus choice following no influence (scrambled graphs)

Area	HEM	Region	*x*	*y*	*z*	*Z* stat	Volume
*Activation in CONTROLS*							
Frontal	L/R	Anterior Cingulate	8	30	30	4.67	165
	L/R	Paracingulate/Anterior Cingulate Gyrus	−4	24	40	4.64	72
	R	Middle Frontal Gyrus/DLPFC	46	10	40	4.92	106
	R	Frontal Pole	32	58	2	5.01	77
	R	Precentral Gyrus	38	2	32	4.65	20
	L	Precentral/Middle Frontal Gyrus	−34	−50	42	5.77	761
	L	Inferior Frontal Gyrus	−54	22	20	4.79	72
Temporal	R	Inferior Temporal Gyrus	54	−54	−8	5.36	254
Parietal	R	Supramarginal gyrus	46	−36	38	4.57	22
	L	Parietal Lobe	−56	−60	28	4.62	42
Occipital	R	Lateral Occipital Cortex	18	−10	56	5.46	973
	R	Precuneus	6	−72	26	5.53	774
	L	Lateral Occipital Cortex	−46	−72	−6	5.39	378
Subcortical	R	Insula	32	24	−6	5.03	98
	L	Insula	−34	18	−4	5.58	233
*Activation in MJ PARTICIPANTS*							
Frontal	L/R	Anterior Cingulate	0	16	54	5.17	642
	R	Frontal Pole	38	52	2	4.93	133
	R	Middle Frontal Gyrus/DLPFC	40	28	30	4.56	103
	R	Frontal Pole	24	48	36	4.50	42
	L	Middle Frontal Gyrus	−46	24	26	4.46	29
	L	Precentral/Middle Frontal Gyrus	−40	2	40	4.98	203
	L	Frontal Pole	−22	52	24	5.10	91
	L	Precentral Gyrus/Inferior Frontal Gyrus	−38	4	26	5.04	81
Temporal	L	Middle Temporal Gyrus	−62	−36	0	4.61	89
Occipital	L	Lateral Occipital Cortex	−24	−68	42	5.90	2516
	R	Lateral Occipital Cortex	26	−68	54	6.05	1648
Subcortical	L/R	Caudate	12	10	10	4.41	29
	R	Insula	34	22	−8	4.82	135
	L	Insula	−30	22	−6	5.65	354
*MJ > CONTROLS*							
Frontal	L	Frontal Pole	28	50	32	2.97	22
Temporal	L	Superior Temporal Gyrus	−62	−34	2	2.86	33
Parietal	L	Superior Parietal Lobule	−22	−58	64	3.42	59

Whole‐brain corrected significant clusters consisted of at least 20 voxels (160 mm^3^) thresholded at *z *=* *2.8 (*P *<* *0.005). HEM represents hemisphere. Coordinates are in MNI space. VOL = volume, in number of voxels (2 × 2 × 2 mm^3^).

#### Activation to choice during congruent versus incongruent decisions (social influence susceptibility)

**Figure 4:**
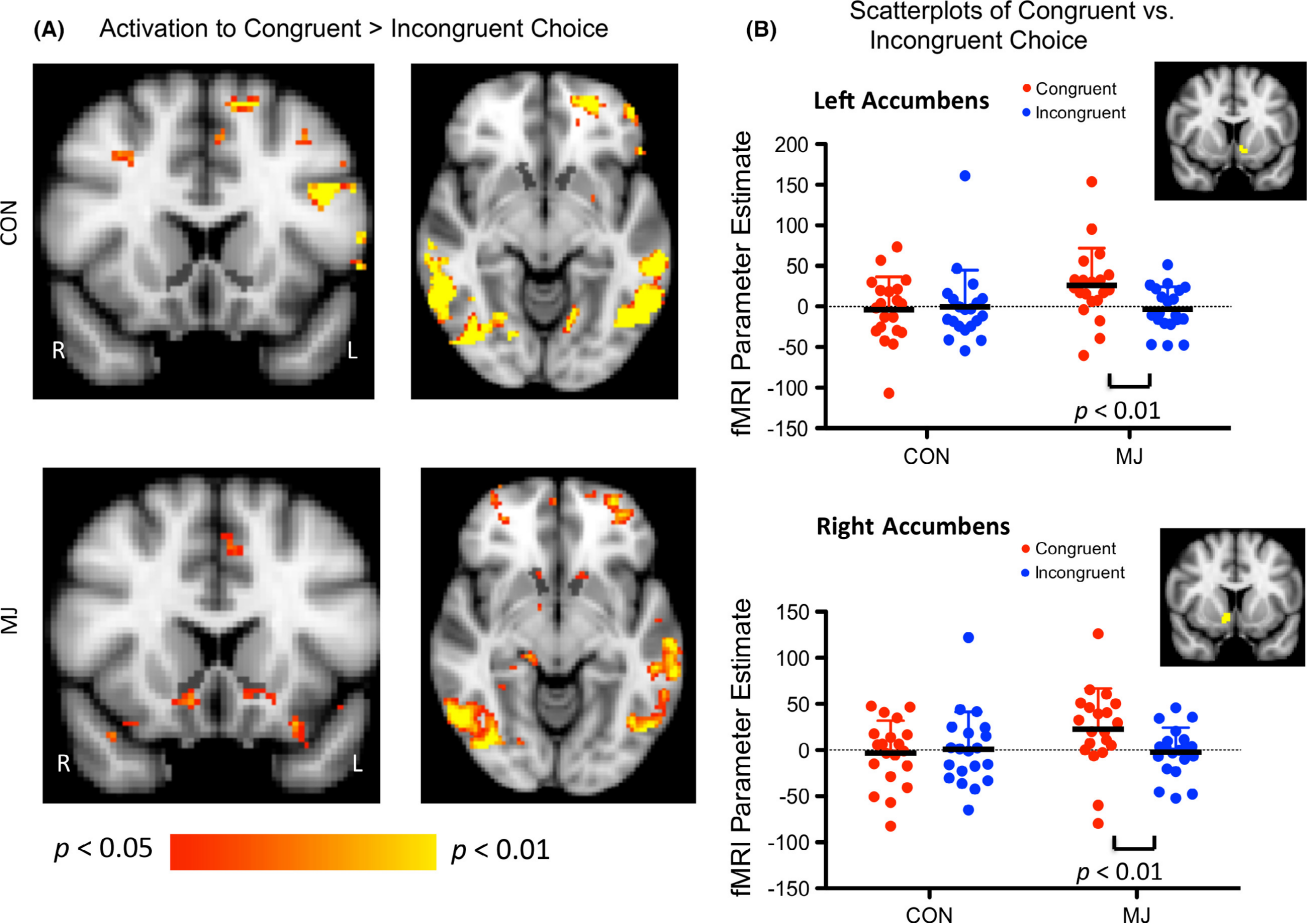
Activation during the choice phase when making congruent versus incongruent choices. (A) Voxelwise comparison of Activation During Congruent versus Incongruent Choices Within Each Group. Anatomical segmentation of the NAc is shown in black. Images are thresholded at *P* < 0.05, uncorrected. (B) ANOVAs between Group and Choice Activation. Segmentation‐based ROIs are shown in yellow. In the left NAc, there was an interaction between group and choice. Subsequent *t*‐tests revealed that there was more activation to congruent than to incongruent choices in MJ, but no significant difference in CON. In the right NAc, there was also an interaction between group and choice. *T*‐tests revealed that there was more activation to congruent than to incongruent choices in MJ, but no significant difference in CON.

This contrast was our primary a priori contrast, and evaluated activity when participants made a congruent choice (i.e., matched the group influence) to activity when they made an incongruent choice (i.e., went against group influence). When we extracted averaged fMRI data (parameter estimates) from the left and right NAc, we observed significant interactions between group and choice bilaterally (Fig. 4A and B). In the left NAc, there was an interaction between group and choice (*F* (1,38) = 6.30, *P* = 0.017) (Fig. 4A and B). Subsequent *t*‐tests revealed that there was more activation to congruent than incongruent choices in MJ (*t* = 3.21, *P* < 0.01), but no significant difference in CON. In the right NAc, there was also an interaction between group and choice (*F* (1,38) = 6.14, *P* = 0.018) (Fig. 4A and B). *T*‐tests revealed that there was more activation to congruent than to incongruent choices in MJ (*t* = 3.01, *P* < 0.01), but no significant difference in CON. As shown in the scatterplots in Figure 4B, MJ showed significantly higher parameter estimates in left and right NAc when subjects followed the group (were congruent with it) than when they did not follow the group (were incongruent with it). The averages of these means shown in Figure 4B, approximate the means of the parameter estimates shown for MJ in left and right NAc in Figure 3B, where combined congruent and incongruent choices are contrasted with choices after scrambled stimuli.

**Figure 5:**
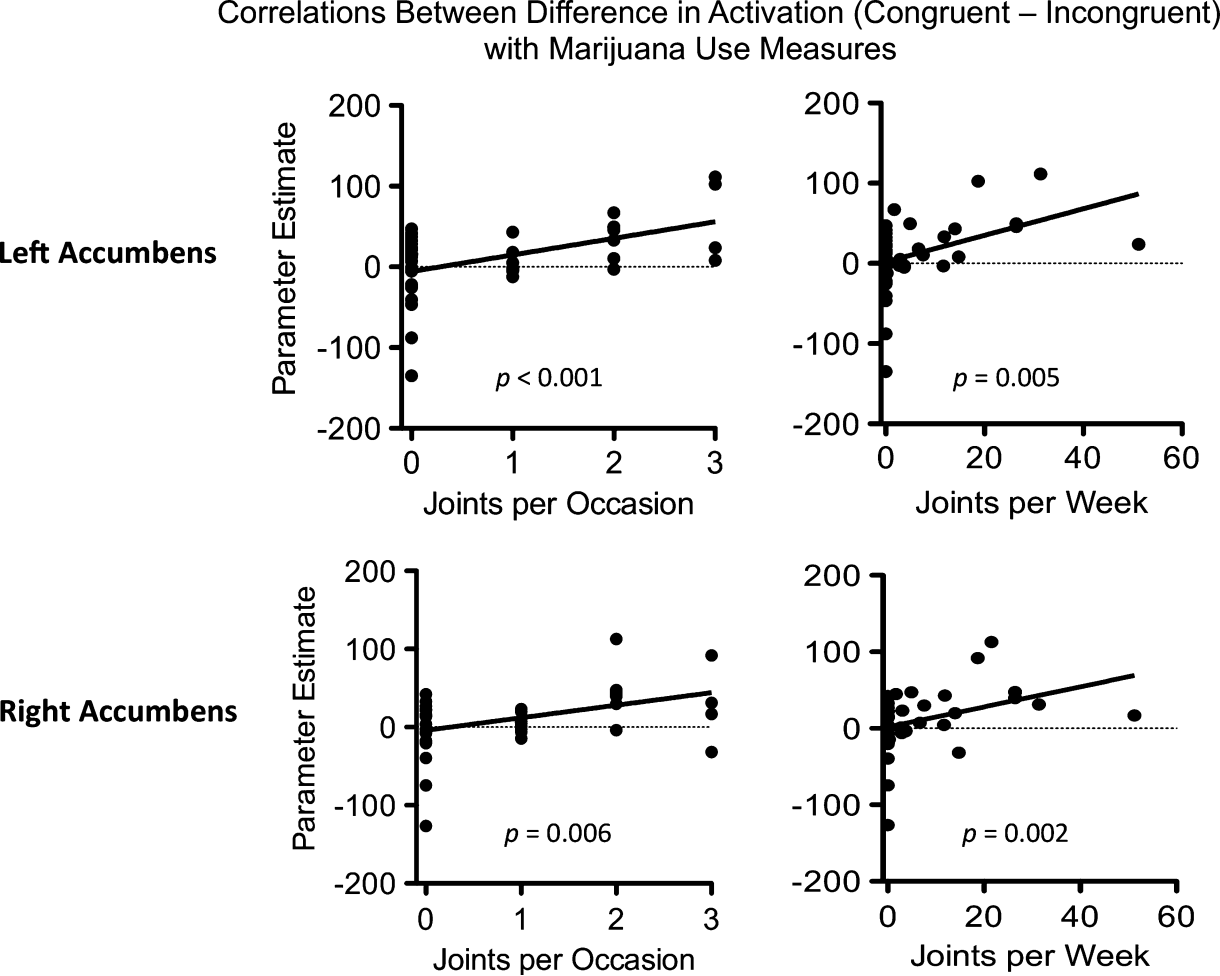
Correlations between activation and drug use measures. Activation in the left and right NAc positively correlated with joints per occasion and joints per week. There were significant associations between neural activation in the left NAc and joints per occasion and joints per week. Activation in the right NAc was also associated with joints per occasion and joints per week.

Activation differences in the left and in the right NAc significantly correlated with joints per occasion (left NAc: *r* ^*2*^ = 0.25, *P *=* *0.001 and right NAc: (*r* ^*2*^ = 0.18, *P *=* *0.006) and joints per week (left NAc: *r* ^*2*^ = 0.19, *P *=* *0.005 and right NAc (*r* ^*2*^ = 0.22, *P *=* *0.002), in that those participants who showed greater NAc activation to congruent choices had greater amounts of marijuana use (Fig. 5). Regression analyses with alcohol use indices were not significant (all *P* > 0.1).

The whole‐brain, voxel‐based analyses revealed that CON and MJ groups showed significantly greater activation to congruent than incongruent choices in several brain regions (Table 3). Both groups showed greater activation to congruent choices in regions such as the bilateral frontal poles, anterior and posterior cingulate, orbitofrontal cortex, middle frontal gyrus, and several temporal plus parietal regions. When MJ and CON were contrasted directly using a voxel‐based analysis with whole‐brain correction, no significant differences were detected.

**Table 3:** Activation to choice during congruent versus incongruent decisions

Area	Hem	Region	*x*	*y*	*z*	*Z* stat	Volume
*Activation in Controls*							
Frontal	R	Frontal Pole	30	64	0	2.87	28
	L	Frontal Pole	−36	58	−2	3.12	428
	L	Inferior Frontal Gyrus	−44	16	26	3.33	407
	L	Precentral Gyrus	−30	−14	52	3.02	404
	L	Middle Frontal Gyrus	−40	4	46	2.90	28
	L	Inferior Frontal Gyrus	−54	26	−2	2.94	26
Temporal	L	Middle Temporal Gyrus	−54	−54	−8	3.43	702
	R	Inferior Temporal Gyrus	56	−52	−10	4.00	615
	L	Temporal Occipital	32	−44	−16	3.42	53
Parietal	L/R	Posterior Cingulate	0	−38	24	3.00	42
Occipital	L/R	Lateral Occipital Cortex	0	−76	14	4.25	6485
*Activation in MJ*							
Frontal	L/R	Anterior Cingulate	−2	22	40	3.07	151
	R	Frontal Pole	22	62	16	3.10	191
	R	Frontal Pole	22	36	−18	3.04	86
	R	DLPFC	28	−2	52	3.61	157
	L	DLPFC	−24	0	52	3.21	220
	L	Frontal Pole	−24	56	20	3.12	152
	L	Frontal Pole	−30	50	2	3.12	266
	L	Middle Frontal Gyrus	−44	32	22	3.09	96
Temporal	L	Superior Temporal Gyrus	−60	−34	0	3.72	351
Parietal	R	Supramarginal gyrus	40	−46	46	2.86	147
	R	Postcentral Gyrus	46	−10	28	2.92	69
	L	Posterior Cingulate	−10	−42	2	3.51	685
Occipital	L/R	Lateral Occipital Cortex	−42	−82	4	4.39	7687
	R	Lateral Occipital Cortex	52	−62	−8	3.43	224
Subcortical	L/R	Thalamus	−2	−8	10	2.80	21
	R	Nucleus Accumbens/Subcallosal cortex^a^	14	18	−12	2.58	10

Whole‐brain corrected significant clusters consisted of at least 20 voxels (160 mm^3^) thresholded at *z *=* *2.8 (*P *<* *0.005). HEM represents hemisphere. Coordinates are in MNI space. VOL = volume, in number of voxels (2 × 2 × 2 mm^3^).

a Met the a priori hypothesis correction.

#### Activation to feedback: notification of points versus no points for monetary reward

**Figure 6:**
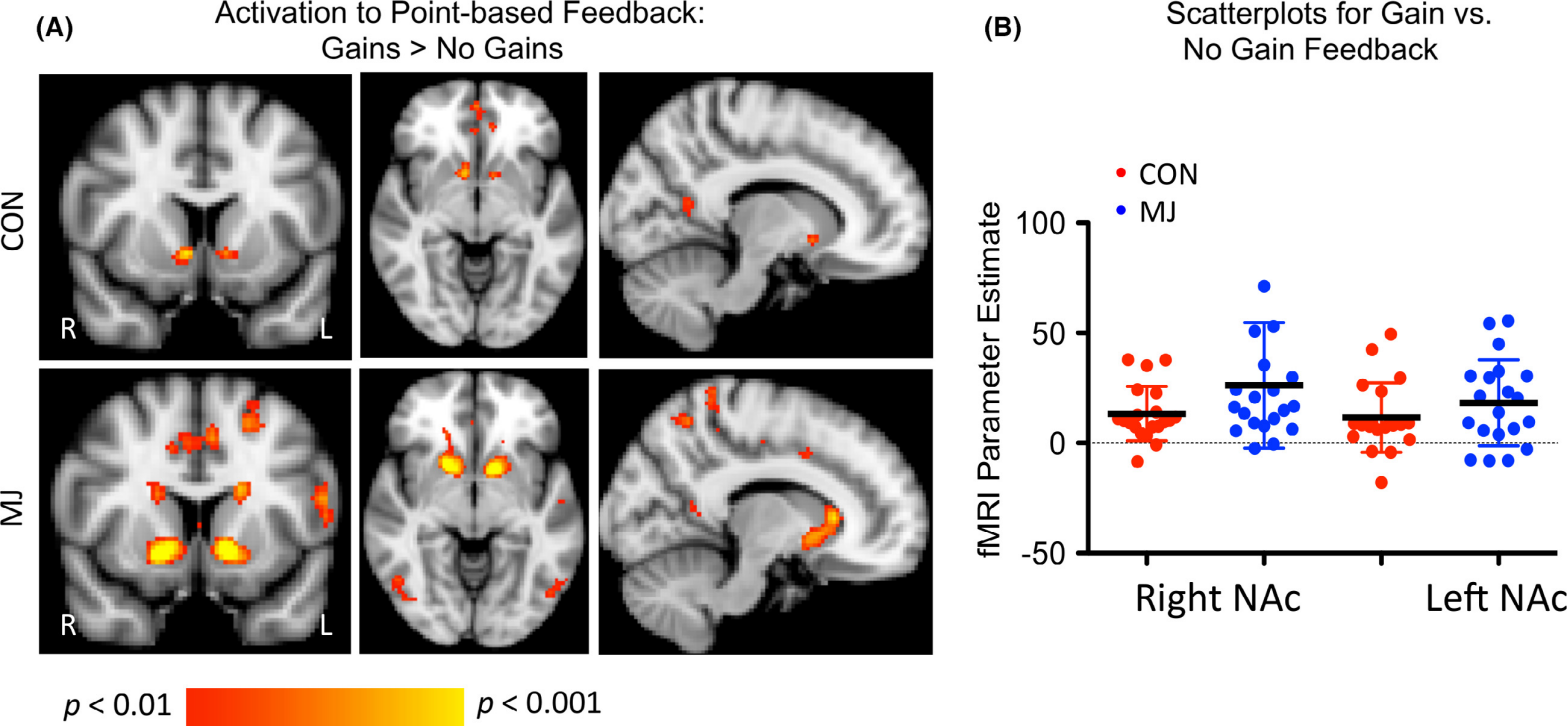
Activation during the feedback phase. (A) ROI‐based Comparison between Gains versus No Gains in the Feedback Phase. Scatterplot of NAc values for the contrast of Gains > No Gains from ROI analysis. There was a trend‐level interaction between group and activation to reward, with MJ showing slightly larger increases in response to gains than controls. (B) Voxelwise Comparison between Gains versus No Gains in the Feedback Phase. Both groups demonstrated significant activation of the NAc during notification of gains. Images are thresholded at *P* < 0.01, uncorrected. The localization of activation during the feedback phase was posterior in the NAc relative to activation related to the choice phase.

This negative control analysis aimed to determine if any group effects during the choice phase reflected general reward processing differences in MJ, or if they might be more specific to following versus going against group influence. When we extracted fMRI data (parameter estimates) from the left and right NAc for the ROI analysis during the feedback phase, we found a trend‐level interaction between group and activation to reward (*F* (1,38) = 2.15, *P* = 0.10), with MJ showing larger increases in response to gains than controls (Fig. 6A and B).

For the voxel‐based, whole‐brain analysis, both groups demonstrated significant signal increases during the feedback phase in the NAc when receiving points compared to not receiving points. The extent of activation volume in the MJ participants in the NAc was larger than that observed in CON, although there were no significant differences in a direct contrast of groups (Table 4). As noted in Table 4, there was also extensive activation in the frontal/parietal cortex. There were no significant effects between marijuana use or alcohol use indices and extracted data from the feedback phase of the task (all *P* > 0.1).

**Table 4:** Activation to feedback: notification of gains versus no gains

Area	Hem	Region	*x*	*y*	*z*	*Z* stat	Volume
*Activation in Controls*							
Frontal	L/R	Medial Frontal Cortex	0	44	−12	3.75	541
	L/R	Precentral Gyrus	2	−24	50	3.21	76
Parietal	L	Precuneus	−6	−58	18	3.55	553
Occipital	L	Lateral Occipital Cortex	−16	−82	40	3.33	180
Subcortical	R	Nucleus Accumbens	8	10	−4	3.44	71
	L	Nucleus Accumbens	−10	10	−4	3.18	25
*Activation in MJ*							
Frontal	R	Precentral Gyrus	46	−12	48	3.85	807
	L	Precentral Gyrus	−42	−14	54	3.96	1648
	L	Middle Frontal Gyrus	−26	26	44	3.32	47
Parietal	R	Precuneus	10	−58	58	3.4	83
	L	Precuneus	−6	−58	60	3.51	292
Occipital	R	Lateral Occipital Cortex	48	−72	6	3.87	283
	L	Lateral Occipital Cortex	−24	−72	46	3.24	307
Subcortical	R	Nucleus Accumbens	16	10	−6	4.03	869
	L	Nucleus Accumbens	−12	8	−6	4.07	162
	L	Caudate	−18	−6	26	3.65	249

Whole‐brain corrected significant clusters consisted of at least 20 voxels (160 mm^3^) thresholded at *z *=* *2.8 (*P *<* *0.005). HEM represents hemisphere. Coordinates are in MNI space. VOL = volume, in number of voxels (2 × 2 × 2 mm^3^).

### Gray matter density

Using anatomical a priori ROIs, GMd differences were observed in the left NAc between groups, with GMd values higher in MJ (*t *=* *2.52, *P *=* *0.016). Differences in the right NAc were not significant (*t* = 1.67, *P* = 0.10).

**Figure 7:**
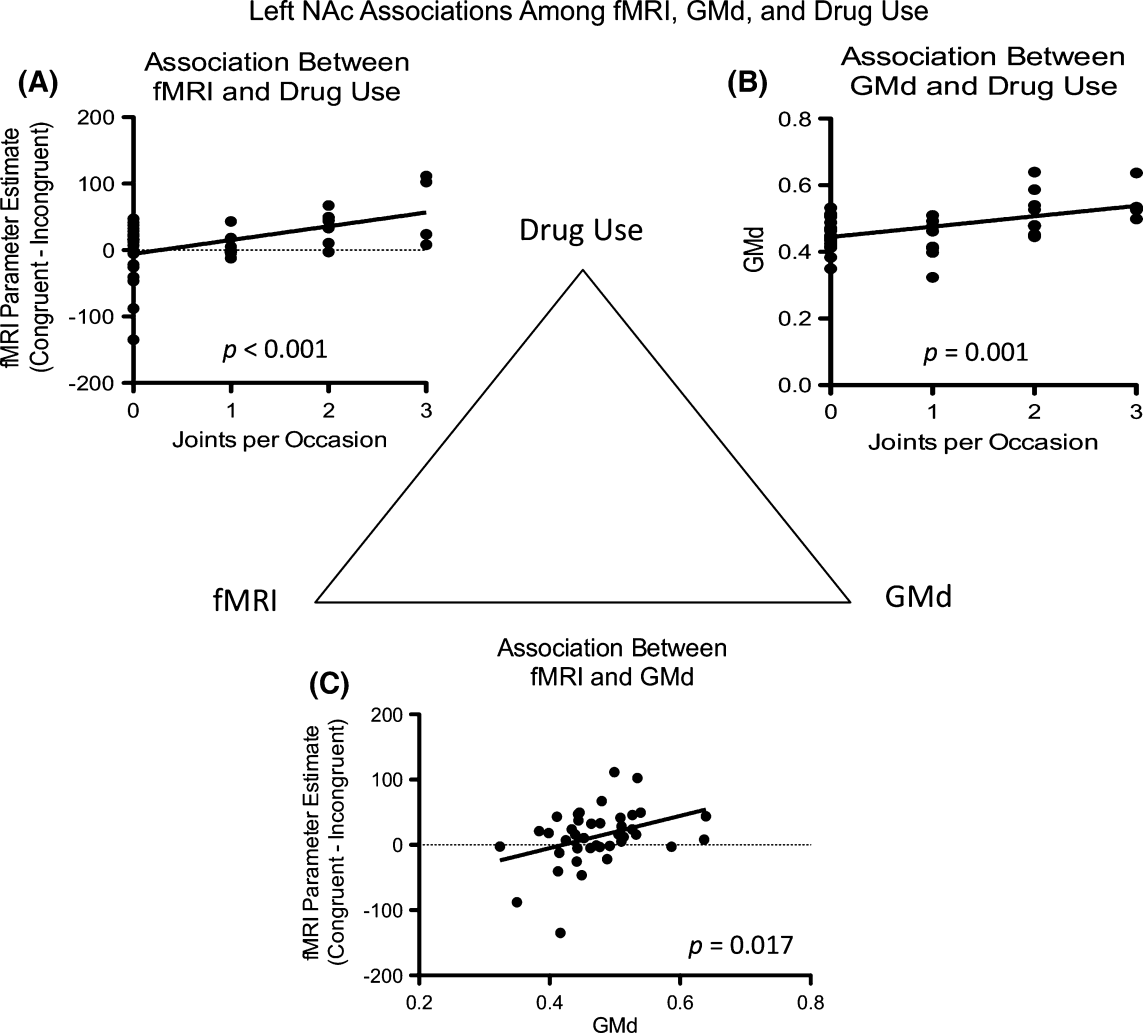
Left NAc associations among fMRI during congruent versus incongruent choices, GMd, and Drug Use, in MJ Participants. There were significant associations between (A) joints per occasion and fMRI activation, (B) joints per occasion and GMd, and (C), fMRI and GMd. GMd in the right NAc was not associated with fMRI measures.

Gray matter density in the left NAc was positively associated both with fMRI response (i.e., parameter estimates) to the contrast of congruent versus incongruent choices (*r* ^*2*^ = 0.14, *P *=* *0.017), and with measures of drug use (*r* ^*2*^ = 0.25, *P *=* *0.001) (Fig. 7). Given 2‐way associations between GMd, fMRI contrast of congruent versus incongruent choices, and measures of drug use were each significant (Fig. 7), a mediation analysis between these three measures was run and found to be not significant.

There were no correlations between fMRI parameter estimates to point‐based monetary reward and either drug use measures or GMd measures.

## Discussion

Although researchers have long‐established the role of social influence in the initiation and maintenance of drug use, few well‐controlled laboratory studies of social influence in decision making have been done in the substance abuse field. This study used a novel neuroimaging task to assess both behavioral susceptibility during decision making, and neural activation to influence. This study had several findings. First, we found that the behavior of the two groups did not differ; both MJ and CON groups were more likely to follow group recommendations than to go against the group. Second, in an a priori ROI‐based analysis of the NAc, we found that MJ activated more strongly to following than not following group influence, and this difference was not observed in CON. Third, activation to point‐based monetary reward in the NAc was not different across groups (though there was a broader expanse of tissue activation in both MJ and in CON relative to the congruent, incongruent comparison). Fourth, activation in the NAc to the following of group influence (as compared to going against) correlated with marijuana use measures and to GMd measures in the NAc; in contrast, activation to point‐based monetary reward in the NAc did not correlate with marijuana use measures, indicating that functional alterations may be specific to social influence. Fifth, whole‐brain, voxel‐by‐voxel analysis revealed multiple other regions implicated in social influence in healthy individuals, such as the cingulate, insula, and regions of prefrontal cortex, were also observed in both MJ and CON participants. One region implicated in social influence, the caudate, produced a number of unique activations in MJ participants that were distinct across the three experimental conditions we evaluated. Namely, middle caudate activation was observed more strongly in MJ than in CON when choices were made after social influence versus after noise images. Further, caudate was not observed to show salient differences between congruent and incongruent choices in either group. Finally, caudate was activated in its dorsal‐most extent in MJ, but not CON, during the feedback phase of the experiment with point‐based monetary reward. These observations, together with the NAc findings, raise the hypothesis that distinct striatal regions (i.e., NAc, middle caudate, dorsal caudate) respond to different aspects of social decision making.

Although we hypothesized that MJ would be more likely than CON to follow group information, data showed that both groups were likely to follow group opinion, specifically during hard trials. A possible reason that we could not detect a difference may have been the large variability within each group (Fig. 2A), which may be accounted for by personality differences in domains other than drug use. We did observe a within‐group difference in reaction time, however, in which CON expended more time on the hard than the easy trials, whereas MJ showed no differences. This could indicate that MJ relied more heavily on the group influence, and therefore spent less time thinking about the hard trials. It could also indicate that MJ were less engaged in the task (although error rates were not different between groups). The MJ group may have made habitual responses without adequate cognitive effort allocated to decision making.

Functional imaging data further revealed significant differences between MJ and CON. For the contrast between choices made after social influence versus no social influence (i.e., combined congruent and incongruent responses vs. noise stimuli), a network of brain structures was activated in both groups, which has been commonly implicated in social cognition. A review article on social cognitive processes reported that the anterior cingulate, anterior frontal poles, and the paracingulate cortex activate to social processes, from self‐reflection to making inferences about others' thoughts (Amodio and Frith 2006); these regions were robustly activated by our line judgment task after participants were presented with group information. Frontal pole involvement is interesting in the context that the frontal poles are believed to play a role in the integration of higher level cognitive processes (Bunge et al. 2009), and dendritic arborization of the neurons in the frontal poles suggest that these regions receive inputs from neurons throughout the prefrontal cortex and other association areas (Jacobs et al. 2001; Ramnani and Owen 2004).

Some of these same regions implicated in social cognition were also observed with the other experimental contrasts. For instance, in the contrast of following or going against group input (i.e., congruent vs. incongruent), we observed anterior and posterior cingulate activation. This is convergent with results from a study by Berns and colleagues (Berns et al. 2010) where adolescents who rated musical clips before and after they learned how their peers had rated them activated the anterior insula and anterior cingulate when they changed their evaluation to match group opinion (Berns et al. 2010). In the contrast of receiving point‐based monetary reward versus no reward in the feedback phase of the experiment, we also saw a distributed network of social cognition regions activated, including dorsolateral prefrontal cortex and dorsal striatum. These observations are consistent with findings from a neuroeconomic game in which participants cooperate or compete with other players, and social decision making is associated with activity in the dorsolateral prefrontal cortex and the dorsal striatum (King‐Casas et al. 2005).

Our finding of dorsal caudate activation during the point‐based monetary reward versus no reward condition has similarities with the King‐Casas et al. findings (King‐Casas et al. 2005), and is intriguing in that it was observed in the MJ subjects alone. This area is involved in multiple features of reward processing, raising the hypothesis that perhaps the MJ group assigned particular value to the social influence information itself. The caudate is a region with a high density of cannabinoid receptors (see (Goodman and Packard 2015) for review), and is involved in a variety of functions, from social reward to goal‐directed behavior, the selection of correct actions, and behavioral control. These observations are intriguing when considering the increased activation in the middle caudate for the MJ group during choice made after social influence, but not in relation to whether they went with, as compared to against, the influence information. This could indicate that the reward regions of the brain may be more responsive to social information in MJ users than among nonusers, or alternatively, that greater recruitment of the caudate is needed to inform the selection of an action during social influence trials in MJ users.

Consistent with such interpretations, the caudate is a structure associated with cooperation in Prisoner's Dilemma games (Rilling et al. 2002). Studies have shown that the magnitude of activation of the caudate can differ across personality characteristics and situational variables. For instance, a study exploring differences between behaviorally inhibited versus behaviorally noninhibited adolescents found greater caudate activation in response to acceptance versus rejection feedback from peers in the behaviorally noninhibited, but not the inhibited adolescents, suggesting that caudate activity may be a marker of social reward in adolescents (Guyer et al. 2014). A study investigating how the quality of peer relationships influenced neural activity showed that chronic peer conflict was associated with greater risk‐taking behavior and heightened activation in brain regions involved in affect and reward processing, such as the striatum and insula (Telzer et al. 2015). Finally, a series of studies showed that during risk tasks such as simulated driving experiments, as well as during nonrisky decision‐making tasks, being observed by peers elicited striatal activation in adolescents, but not among adults (Chein et al. 2011; Smith et al. 2015), supporting the idea that the striatum is sensitive to social context, and can be linked to individual differences in sensitivity to peer influence.

In contrast to these activation profiles for the middle caudate and dorsal caudate, we observed a different set of activation profiles for the ventral most part of the striatum, or NAc. When we contrasted activation during congruent versus incongruent choices, both groups demonstrated activation in a variety of frontal and temporal regions, but only MJ demonstrated greater activation during congruent compared to incongruent choices in the NAc. The NAc has also emerged as an important region in social neuroscience (Aharon et al. 2001; Blood and Zatorre 2001; Knutson and Wimmer 2007), in addition to reward processing more generally (Becerra et al. 2001; Breiter et al. 2001; Tom et al. 2007; Zaki et al. 2011) and activation in this study replicates earlier research suggesting that cooperation/agreement involves this region (Klucharev et al. 2009; Zaki et al. 2011). It is intriguing that the NAc activation during congruent responses (perhaps reflecting intrinsic reward) was seen only in MJ participants. Unlike the facial attractiveness tasks, which reflect on a person's preferences, the line judgment task was objective. It is possible that CON did not find agreement with the group particularly rewarding on such a task. Berns et al. (2005), using a mental rotation task, also did not find NAc activation when control participants agreed with group consensus (Berns et al. 2005). The heightened NAc activity to congruent responses in MJ participants may reflect hyperactivity of reward circuitry in social decision making. Intriguingly, individuals who reported using more marijuana also showed greater activation to congruent versus incongruent choices, demonstrating that the neural processing of social influence may differ among users with different patterns of use.

Previous studies have shown that NAc function is altered in cannabis users. Magnitude of NAc activation to MJ cues has been shown to correlate with more use‐associated problems, as measured by the marijuana problem scale (Filbey et al. 2009); it is possible that a number of items on this scale relate directly or indirectly to social interactions (e.g., problems with family or job loss), consistent with the altered NAc function during social decision making in this study. NAc function has also been shown to increase during receipt of money loss during a MID paradigm in cannabis users (Yip et al. 2014), which cannot be directly compared to the current results, but raises the importance for future studies evaluating both gains and losses from the outcome of an objective judgment task.

During the feedback phase (point‐based monetary reward), we observed robust NAc activation in both groups during gains compared with no gains. NAc activation in the feedback phase did not correlate with drug use measures. These findings contrast with the significant group by choice interaction of NAc activation during the choice phase of the social influence task, as well as with the correlation between this NAc activation (for congruent vs. incongruent choices) and marijuana use measures. This apparent contrast in results regarding correlation with MJ use across phases suggests that differences in NAc activation between groups may be specific to deciding to follow or not follow social influence; further work is needed for confirmation. A possible interpretation is that with recreational marijuana use, NAc activation to social influences is more highly engaged during the making of choices (i.e., deciding to take actions to engage in drug use) than during the processing of consequences of those actions.

These findings raise the question of the relevance of activation differences between MJ and CON in the absence of behavioral differences. To use fMRI as a tool to explain brain function in a contrast‐based paradigm such as the one used in this study (e.g., as opposed to a parametric design), it is important that neural activation be unconfounded by behavioral performance in order to clearly interpret fMRI findings. Many cross‐sectional fMRI studies have intentionally minimized differences in task success or behavior to avoid a confounding group difference (e.g., (Beck et al. 2009; Bjork et al. 2012, 2008b; Wrase et al. 2007). This has been an issue in substance abuse literature, where the matching of behavior or of brain measures is considered imperative for interpretation of differences in the other measure (Tapert et al. 2007; Bjork et al. 2008a). Furthermore, brain activation differences without behavioral differences have been interpreted as reflecting efficiency in cognitive processing in the service of normal performance (Sullivan and Pfefferbaum 2005). Neural differences, with intact behavioral performance, may be a covert marker of neural inefficiency in the context of processing social influence in marijuana users.

Finally, our post hoc analysis of GMd in the NAc was designed to explore whether structural alterations were related to fMRI activation during the choice phase in MJ users. We did not observe any mediation effects, but we did observe a triangulation among drug use measures, GMd in the NAc, and fMRI activation in the NAc to social influence. Such a finding was not observed with activation to point‐based monetary reward. This interaction suggests the potential for future studies to assess how NAc structure might affect decision making in MJ users. Furthermore, longitudinal studies can assess whether marijuana exposure is a causal factor leading to the observed differences in the structure and/or function of the NAc. It should be noted that recent studies suggest that the T1 signal, used to extract VBM measures, may be affected by transient blood flow (Lu et al. 2004; Salgado‐Pineda et al. 2006; Franklin et al. 2013), and therefore further investigation of these findings evaluating blood flow versus structure may be a fruitful avenue of investigation.

There are several caveats to this study. First, since our task did not detect a behavioral difference between groups, this study does not comment on whether MJ may be more susceptible to peer influence than nonusers, or may be at greater risk for further drug use in social settings. Future studies can assess whether MJ users are more susceptible to influence in specific environments that foster drug use. It would be particularly intriguing to assess whether those MJ users with greatest NAc activation to congruent versus incongruent choices were also most likely to increase marijuana consumption in social settings. Furthermore, since the task in this study depended on deception (i.e., the participants were told that the responses presented were those of “previous participants”), different levels of “belief” may have modulated behavioral choices or neuroimaging results. Future iterations of this and other social influence paradigms may need to have participants rate the believability of the paradigm on a numerical scale to investigate whether a relationship exists between behavior/neural activation and believability. Second, as with most imaging studies, this study does not address cause and effect; neural activation to group agreement in the NAc may develop as individuals use drugs, and chronic drug use may influence decision making. This study generates hypotheses for future work addressing causality. Third, we did not match groups as tightly on alcohol or cigarette use as on other demographic measures (though dependence was exclusionary, and alcohol metrics did not correlate with the fMRI findings), which may have contributed to our results and are important covariates for future work. Fourth, our sample size of 40 participants does not allow us to investigate higher order interactions such as personality characteristics, gender, or other variables that may affect social influence susceptibility. Finally, the significant ROI interaction in the NAc between group (MJ, CON) and choice (congruent, incongruent) and relationship of activation to MJ use measures support the hypothesis that MJ users show greater involvement of the NAc and reward regions in social decision making. However, as with any single imaging study, the finding in the NAc should be replicated and further investigated in future studies.

Identifying brain regions that differ in response to social influence may guide treatments such as lifestyle management, which could encourage marijuana users to find alternative positive reinforcement in the natural environment. An example of such a treatment is the Community Reinforcement Approach, which encourages involvement in nondrug‐related pleasurable social activities (Meyers et al. 2011). Future studies are needed to determine whether patterns of activation to social influence may be a potential treatment target, and whether distinct striatal regions (i.e., NAc, middle caudate, dorsal caudate) responding to different aspects of social decision making can be harnessed for such treatment.

## Conflict of Interest

The authors declare no competing financial interests.
